# Upgrading or Polarizing? Gendered Patterns of Change in the Occupational Prestige Hierarchy Between 1997 and 2015

**DOI:** 10.3389/fsoc.2022.834514

**Published:** 2022-04-11

**Authors:** Ylva Ulfsdotter Eriksson, Tomas Berglund, Erica Nordlander

**Affiliations:** ^1^Department of Sociology and Work Science, University of Gothenburg, Gothenburg, Sweden; ^2^Department of Social Studies, Linnaeus University, Växjö, Sweden

**Keywords:** occupational change, polarization, upgrading, occupational prestige score, gender segregation

## Abstract

This article contributes to the discussion on how the Swedish labor market is changing: is it upgrading or polarizing? Drawing on *the Swedish Labor Force Survey* the study examines the overall changes in the occupational job structure in Sweden by exploring how women and men were distributed within the occupational prestige hierarchy at two points of time, 1997 and 2015. The results show that changes in the labor market have resulted in different patterns of how women and men are distributed within the occupational prestige hierarchy. Women have an upgrading movement and have entered high-prestige occupations, while men have been subjected to job polarization, with an increase in employment in low-prestige occupations, as well as high-prestige ones.

## Introduction

This study examines the overall changes in the occupational job structure in Sweden by exploring how women and men were distributed within the occupational prestige hierarchy at two points of time, 1997 and 2015. Occupational prestige is a symbolic measure of occupations social standing based on desirability (cf. Carlsson, 1958; Reiss, 1961; Treiman, 1977; Oesch and Piccitto, 2019) and is thus a significant factor of stratification in society. Occupational prestige is also a convenient measure to use in the study of changes in the occupational structure since previous research has repeatedly shown that the occupational prestige hierarchy is stable over time (Carlsson, 1958; Treiman, 1977; Nakao and Treas, 1994; Svensson and Ulfsdotter Eriksson, 2009). That occupations are ascribed the same prestige over time, contributing to the stability of the occupational prestige rank order, has been referred to as the “Treiman constant” (Hout and DiPrete, 2006, p. 3). Consequently, by focusing on occupational prestige, it is possible to discern where job growth in the occupational hierarchy takes place.

The relationship between gender and occupational prestige is not clear. It is commonly stated that men have the “best” occupations and dominate in high-prestige occupations (cf. Charles and Grusky, 2005). However, as shown by England (1979), except for the most prestigious occupations, women and men were quite equally distributed between the different prestige groups. Still, as shown in other studies, the relationship between gender and occupational prestige is not clear (cf. Ulfsdotter Eriksson, 2006; Magnusson, 2009; García-Mainar et al., 2018). Thus, by taking the gender-segregated labor market into account, this study acknowledges that changes in the job structure may have affected women and men differently with regards to how they are distributed within the occupational prestige hierarchy.

Research widely agrees that the new digital technology has had a pervasive impact on Western labor markets in the last decades (Brynjolfsson and McAfee, 2014; Frey and Osborne, 2017). However, there is less agreement on how these changes have affected the occupational structure. Two general lines of argument are identified: The first strand of research claimed that the increased use of digital technology replaces low-skilled labor and enhances the productivity of high-skilled workers. This is described as an upgrading of the occupational structure with a decrease in the lower-end and raising employment in high-skilled occupations (Katz and Murphy, 1992; Berman et al., 1998; Acemoglu, 2002). The second strand of research stated that the new technology replaces people in routine-based occupations mainly positioned in middle-level jobs, and increases the number of employees in both low and highly skilled non-routine occupations, resulting in a polarization of the occupational job structure (Autor et al., 2003; Goos and Manning, 2007; Acemoglu and Autor, 2011).

In the European context, a great variation of tendencies has been discerned. Both Germany and the United Kingdom have shown polarization tendencies in the last decades (Dustmann et al., 2007; Goos and Manning, 2007), while Murphy and Oesch (2018) showed a general pattern of upgrading in Ireland and Switzerland. Consequently, in comparative European studies, diverging patterns of both upgrading, polarization, and even down-grading, are found (Fernández-Macías, 2012; Eurofound, 2017).

The debate on the impact of new technology on occupational change is present also in Sweden. According to Åberg (2003), the upgrading pattern that for a long time characterized the Swedish labor market changed in the 2000s in a polarized direction (cf. Åberg, 2015; Adermon and Gustavsson, 2015; Heyman, 2016). Another study, covering the period between 2000 and 2015, showed tendencies of polarization, with neither growth nor decline in the lowest-paid occupations, a decline in middle-level jobs, and still a strong growth in the highest-paid sections of the occupational structure (Berglund et al., 2020a). However, findings like these are questioned by researchers who argue that polarization is a myth, claiming that the Swedish labor market continuously is upgrading (Oesch and Piccitto, 2019; Tåhlin, 2019).

Important to note is that these different strands of research, based their evidence on different variables: Where Tåhlin used job qualification, measured by educational requirements, to show upgrading, Åberg and Berglund and authors used changes in the job-wage structure to argue for polarization. Oesch and Piccitto (2019) used different indicators to study changes in job quality over time; besides median wages, job satisfaction, and average educational level, they also included a measure of occupational prestige. All these indicators showed that the occupational structure in Sweden moves in the direction of upgrading (Oesch and Piccitto, 2019). The inclusion of occupational prestige is an interesting move in the study of changes in the occupational structure. Prestige is a multidimensional measure, substantiated by wage, education, skills, and power, and as an expression of social reputation, it also describes “good” and “bad” jobs in a complex way (cf. Carlsson, 1958; Treiman, 1977; Ulfsdotter Eriksson, 2006; Oesch and Piccitto, 2019).

The present study returns to the same period as Oesch and Piccitto (2019) to study changes in the occupational structure from the perspective of occupational prestige. In so doing, it contributes with additional knowledge and new aspects of the restructuring of the occupational job structure: Firstly, by focusing on occupational prestige, this study adds to research on an important piece of the very complex puzzle that these changes in occupational structure constitute. Secondly, by using the Swedish national labor force survey (LFS) an unchanged classification of occupations (SSYK 96) have been possible to use for the whole period [Oesch and Piccitto (2019) needed to crosswalk between two different occupational classifications when using the European LFS]. Thirdly, we conduct a decomposition analysis of the mean change of occupational prestige, thereby being able to identify the main drivers of change moving the distribution of employees in the prestige hierarchy. Fourthly, and most importantly, this study contributes by considering gender. This is relevant for two reasons. First, the Swedish labor market is characterized by vast gender segregation allocating women and men into different occupations and sectors (Nermo, 1999; Charles and Grusky, 2005). Technological changes may thus have affected the job structure for women and men in different ways. Secondly, it is commonly argued that “the best occupations will be dominated by men” (Charles and Grusky, 2005, p. 8), but as shown by England (1979), women and men were evenly distributed across the occupational prestige hierarchy, except for the most prestigious occupations. Still, changes in the job structure may have affected the distribution of women and men in the prestige hierarchy.

The aim of this study is thus to examine the overall changes in the occupational job structure in Sweden by exploring the occupational prestige structure in Sweden at two points of time, 1997 and 2015. More specifically, the study (1) explores how employed women and men are distributed vertically within the occupational prestige hierarchy; (2) describes how the distribution of women and men has changed vertically and horizontally between the year 1997 and 2015; (3) explains changes in occupational prestige mean score between the two points of time and in relation to gender.

This rather elaborated introduction is followed by a section in which occupational change, prestige, and gender segregation in the labor market are further discussed through the literature. Thereafter follows a note on methods. The empirical findings are presented in three sections following the research questions addressed. The article ends with some concluding remarks.

## Occupational Change Indicated by Occupational Prestige

Digitalization, with increasingly advanced technology, computers, and robots, affects tasks previously conducted by humans (Frey and Osborne, 2017), which is also believed to have a profound impact on the occupational structure (Brynjolfsson and McAfee, 2014). Digitalization entails two concurrent processes of occupational changes. *Skill-Biased Technological Change* (SBTC) asserts that low-skilled workers are substituted whereas the productivity of high-skilled work is augmented by digital technology. This *upgrading* was observed in the United States 1970–1990, when the number of low-skilled workers decreased, while the number of high-skilled increased (Katz and Murphy, 1992; Berman et al., 1998; Oesch, 2013).

More recently, a *polarization* of the occupational structure has been observed. According to the theory *Routine-Biased Technological Change* (RBTC), digital technology enhances productivity in cognitive tasks with non-routine character and replaces routine work tasks, while manual non-routine tasks remain more or less untouched. Autor et al. (2003) showed that in the United States between 1990 and 2000, low-paid, low-skilled jobs increased, as did high-paid, high-skilled jobs, while the routine jobs in the middle of the occupational structure decreased and were replaced with digital technology (see also Goos and Manning, 2007).

Oesch and Piccitto (2019) stated that the dominant focus on wages were insufficient to explore changes in the job structure to determine whether it is polarizing or upgrading. They studied changes of “good” and “bad” jobs in the occupational structure through four different indicators: earnings, skill requirements, job satisfaction, and prestige. Drawing on Treiman (cf. 1977), they argued that ‘Prestige is a measure of an occupation's social desirability and thus taps into symbolic rather than economic power' (Oesch and Piccitto, 2019, p. 449). Their comparative analysis showed upgrading tendencies for Sweden, and they rejected the polarization thesis.

Sweden is an interesting case to study since scholars using different measures arrive at different conclusions. The Swedish labor market has long been characterized by upgrading. From 1974 to 2000, jobs with low qualifications decreased and more qualified jobs increased (Korpi and Tåhlin, 2011). In a more recent publication, Tåhlin (2019) showed continuous upgrading, which is in line with the findings of Eurofound (2017) and Oesch and Piccitto (2019). Yet, according to Åberg (2015), the Swedish labor market moved toward wage polarization from 2008 and 2012, with an increase in low- and high-paid jobs, while middle-layer jobs decreased in number (cf. Adermon and Gustavsson, 2015; Heyman, 2016; Berglund et al., 2020a).

### Occupational Prestige Hierarchy

Against the background of the contradicting findings in the Swedish case, showing both upgrading and polarization, this study argues for the necessity of an additional measure and thus uses prestige to explore changes in the occupational structure.

Occupational prestige is a symbolic measure of occupations' social standing based on desirability (cf. Carlsson, 1958; Reiss, 1961; Treiman, 1977; Oesch and Piccitto, 2019). It is a complex notion and the measures used in studies of changes of the job structure, such as wage, skills, and qualifications, also substantiate occupational prestige. Occupational prestige has been described as a function of income, education, skills, and power (Svalastoga, 1959; Marsh, 1971; Treiman, 1977; England, 1979; Svensson and Ulfsdotter Eriksson, 2009).

Occupational prestige is a means to classify and rank order occupations in terms of social standing (Reiss, 1961; Ganzeboom and Treiman, 1996). Previous research has shown great stability in the occupational prestige hierarchy within and between countries and over time (Treiman, 1977). Drawing on prestige studies from 55 countries, Treiman (1977) developed generic prestige scores for over 500 occupational titles and constructed the *Standard International Occupational Prestige Scale* (SIOPS: cf. Ganzeboom and Treiman, 2010). The correlation between the SIOPS and later measurements of occupational prestige is high (cf. Nakao and Treas, 1994), applying also to the Swedish occupational prestige hierarchy (Svensson and Ulfsdotter Eriksson, 2009).

The prestige scale has been described as “the only universal sociologist has discovered” (Hout and DiPrete, 2006, p. 3), making it ideal for comparative research (Härkönen et al., 2016). Since this study aims to compare the occupational job structure between two points in time and compare outcomes of changes for women and men, occupational prestige seems to be an ideal measure. In addition, Härkönen et al. (2016) argued that prestige indicates women's occupational attainment more precisely than socioeconomic indexes since women have higher educational levels, but lower wages than men have (Warren et al., 1998).

### Prestige and Gender Segregation on the Labor Market

A key issue in the present study is whether changes in the job structure, measured by occupational prestige, have affected women and men differently. It is a reasonable assumption because of the sexual division of labor, allotting women and men into different occupations both horizontally and vertically within the job structure. Charles and Grusky (2005) argued that the reproduction of gender inequalities in the labor market have two dynamics: The *horizontal dynamic* segregates men and women into different occupations, while the *vertical dynamic* allots men to the more prestigious ones (cf. Emerek, 2008; Ridgeway, 2011).

The Swedish labor market is vastly gender-segregated (Nermo, 1999; Charles and Grusky, 2005). The horizontal segregation is extensive and only 15 percent of the women and 14 percent of the men work in occupations defined as gender-equal at a 40/60-percent share of the sexes (Statistics Sweden, 2018). The main explanation of the strong gender segregation on the Swedish labor market is the expansion of the public sector and welfare jobs in the 1970s and 1980s, which both created possibilities for women to enter the labor market (e.g., child-care elderly and personal care) and offered jobs within the sector (Esping-Andersen, 1990). In 1997, about 37 percent of all employees were public employed—in 2015 this had declined to 32 percent, although still a large public sector in comparison to most OECD countries. Within the public sector, the vast majority are women −73 percent in 1997. Consequently, the most common occupations for women are preschool teachers, childcare workers, and assistant nurses, and for men, carpenters, and electricians.

Vertical segregation refers to the hierarchical aspects of the division of labor and may concern either managerial positions or matters of social stratification and prestige. Regarding the former, in Sweden, women hold 39 percent of the managerial positions, more so in the public sector (65%) than in the private (31%) (Statistics Sweden, 2018). In terms of prestige, a previous study of Sweden showed that among the 20 highest rank-ordered occupations, 12 were male-dominated, 3 were female-dominated and 5 were gender-balanced (Ulfsdotter Eriksson, 2006). Still, Magnusson (2009) showed that the proportion of women did not affect an occupations' prestige. In a more recent study from Spain, García-Mainar et al. (2018: 355f) showed an inverted U formed relationship, where “Occupations in the upper tail of the distribution (the most socially recognized) display higher average female shares than those in the first seven deciles, indicating that occupations with greater occupational prestige are those where the shares of women and men are more similar.”

Still, more recent data showed that in both the vertical and the horizontal gender segregation in the Swedish labor market is decreasing (Halldén, 2014; Kjellsson et al., 2014), and at a faster rate than in other European countries (Emerek, 2008). Today, women dominate in higher education (Statistics Sweden, 2018), and are increasingly entering higher white-collar occupations such as physicians and judges (cf. England, 2010). The share of women in the elite- and top-wage positions are growing and women with the appropriate education have the potential to outcompete men (Bihagen et al., 2014). Higher education has been “at the nexus of status-competition and status equality efforts” (Bradley, 2000, p. 10). Therefore, the expansion of white-collar occupations may be favorable for women and open new possibilities for highly educated women. Women are now to a further extent in a majority in some high-prestige educational programs. However, as pointed out by Bradley, education is strongly sex-segregated, and men still prevail in some fields that lead to high-prestige occupations.

## Data and Methods

The empirical analysis draws on quantitative data from *the Swedish Labor Force Survey* (LFS), collected by Statistics Sweden to produce official statistics on employment and unemployment. The population is sampled each quarter, including ~60,000 respondents (in three separate samples in each of the 3 months of the quarter). The LFS consists of a panel in which respondents are interviewed eight times over eight quarters (2 years). One-eighth of the panel is replaced each quarter with a new so-called rotation group.

The present study focused on employees (i.e., dependent employment) 16–64 years and used the yearly sample for 1997 and 2015 for the descriptive analyses, with 177,144 observations in 1997 and 192,114 in 2015. To be included in the sample, the individual needed to work at least 1 h during a reference week. The observations include a maximum of four observations of each individual. In the descriptive analyses, official weights from Statistics Sweden are used to correct for biases in the sampling, the dependence of the observations (the same individual may reappear a maximum of four times per year), and to recalculate numbers to population parameters (that is, absolute population numbers). In the regression analyses, only the newly recruited first rotation group in LFS was used for each month of the year. In this way, the observations become independent (i.e., an individual are only interviewed one time each year), which is an important requirement for ordinary least square regressions. Rotation group 1 is similar in size in each month of the year, however, the number of yearly observations becomes fewer in these analyses (21,214 in 1997 and 23,461 in 2015) compared to the descriptive analysis of the total sample.

### Indicator of Occupational Prestige

The main dependent variable in the analyses was *occupational prestige* indicated by the SIOPS, which ranges from 13 to 78. The correspondence between SIOPS and the Swedish prestige hierarchy is high (Svensson and Ulfsdotter Eriksson, 2009), which was also confirmed in a recent study (Nordlander and Eriksson, 2022). SIOPS-scores were imputed to each occupation on the 4-digit level (Ganzeboom and Treiman, 2010). We conducted a descriptive analysis of the distribution by defining quintiles of the prestige distribution. To construct the quintiles, we first arranged the occupations from the lowest to the highest prestige, and frequencies of observations in occupations with different prestige were calculated. The quintiles were defined for the 1997 data by dividing the prestige hierarchy into five groups of equal size, from the lowest prestige (quintile 1) to the highest prestige (quintile 5). The cut-off points of the 1997 quintiles were then used to recalculate the prestige groups in 2015. Changes in the number of employees in occupations with different prestige could thus be analyzed. In the decomposition analysis (see below), the SIOPS-scale was used as the dependent variable with the focus of explaining overall changes in mean prestige for employed distributed in the occupational structure.

### Analysis

To explore the distribution of prestige on a sex-segregated labor market in times of occupational change, the statistical analysis was conducted in two steps. Firstly, the percentage change of the numbers employed in prestige quintiles in 1997 and 2015 was analyzed, including a separate analysis of women and men, as well as descriptives of the main occupational groups within the quintiles.

Secondly, a decomposition analysis of the mean change of occupational prestige (SIOPS) was conducted. The statistical technique was Blinder-Oaxaca decomposition (Blinder, 1973; Oaxaca, 1973; Jann, 2008). It specifies how much compositional changes in the independent variables account for the mean change in the dependent variable (prestige), making it possible to discern what factors that mainly explain changes in mean occupational prestige. In the analysis, the changes were decomposed into an explained and an unexplained part. The former depends on changes in the composition of the independent variables; for example, if the share of women in employment has increased over time. To estimate the explained part, regression coefficients of a pooled sample from the years 1997 and 2015 were used. This was conducted in Stata using the Oaxaca-command with the pool-option (Jann, 2008). By using the detail option, the amount explained by each of the included variables was specified. The unexplained part had different characteristics. Some were unmeasured heterogeneity hidden in the intercept of the regressions. However, unexplained differences due to changes in the impact of the independent variables are shown; for example, if the impact of sex (the size of the coefficient) has become stronger over time. The latter informs that additional processes are taking place that is not accounted for by compositional changes in the independent variables. The presentation of the analyses includes the change in the mean value of prestige, the composition of the independent variables at the two time-points, their respective regression coefficients in 1997 and 2015, and for the pooled sample, as well as the amount of the difference explained (and unexplained) by each of the independent variables. Separate analyses were conducted for the sexes, as well as the higher and lower half of the prestige distribution. The latter was due to the polarized pattern of change that the descriptive analysis revealed.

Two central independent variables of the decomposition analysis were *Sex* (men/women) and the *Occupational wage structure* (OW-structure), which was a key variable for the analysis as it has been used to measure in which direction the occupational structure moves when it comes to wage and skill levels, i.e., upgrading or polarization (Autor et al., 2006; Åberg, 2015). We imputed wages per occupation on the 3-digit level based on the Swedish Wage Structure Statistics referring to the occupation's median full-time wages for the year 2013. No agreement exists of which year over a period to use when calculating the OW-structure. Both the mean inflation adjusted wage for a whole period (Åberg, 2015) as well as a single arbitrary year (e.g., Eurofound, 2017) has been used. One argument of using the last year is that the processes of technological change believed to be at work – SBTC or RBTC – may have changed the wage level of the occupations. However, the ranks of the occupations seem rather stable as they between 2000 and 2013 correlate strongly (*r*^2^ = 0.92) (Berglund et al., 2020b).

Several control variables relevant to various labor market outcomes were also used in the decomposition analysis: *Age* (16–64 years) is an important factor of stratification and there are signs of age discrimination on the Swedish labor market, in that it is harder for older people to find new employment in comparison to young applicants (Ahmed et al., 2012). *Education* (below tertiary (below 4 in ISCED 97)/tertiary (4 and above in ISCED 97)) has become an increasingly important factor and women dominate in higher education (Statistics Sweden, 2018). Education is closely intertwined with prestige, why it was important to control for (Treiman, 1977). S*ector* (private/public) has been an important structuring factor in Sweden, and during the time-period 1997–2015, about one-third of the workforce was employed within the public sector, and the vast majority were women (Statistics Sweden, 2018). Finally, as immigrants experience disadvantages in terms of education and labor market outcomes (Le Grand and Szulkin, 2002; Rydgren, 2004), the *country of birth* (native/foreign-born) was also controlled for.

## Findings

### Changes in the Number of Employees in Five Prestige Groups in 1997 and 2015

**Figure 1:**
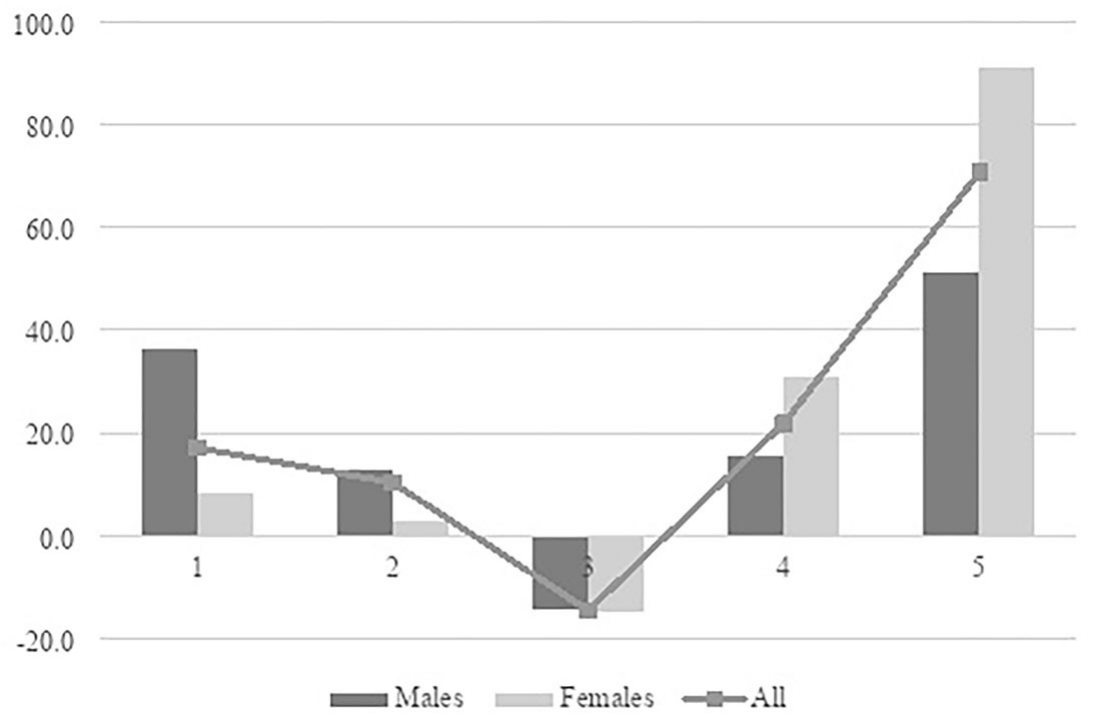
Percentage change between 1997 and 2015 in the number of employed in quintiles of occupational prestige. Weighted LFS-data.

Figure 1 (below) describes both the overall pattern of change of the number of employees in the five prestige groups between 1997 and 2015 and separate growth rates for women and men. The solid line, describing the overall pattern of employment in different prestige groups, shows tendencies of polarization: The two low-prestige quintiles increased slightly (17 and 10% in growth), the middle prestige strata decreased by 15 percent, and the two high-prestige groups displayed an upward trend, indicating that employment in high-prestige occupations has increased over time (22 and 71%, respectively). As the sample includes individuals with potentially very few working hours, we tested to recalculate the distributions with employees having a working week of 20 h or more. However, the results became very similar as above. Consequently, our results show a clearer tendency of polarization than what Oesch and Piccitto (2019) found for Sweden. Thus, the overall pattern of changes in the occupational prestige structure is in line with the polarization of the so-called occupational-wage structure shown by Åberg (2015).

However, the changes have affected women and men differently, as illustrated by the bars in the figure. For women, the growth in high-prestige occupations clearly outweighs the growth in low-prestige jobs. The growth in prestige group 5 was 91 percent for women and 51 percent for men, and the increase in prestige group 4 was 31 respective 15 percent. The number of men has increased more than the number of women in the two low prestige groups (36% and 12% compared to 8 and 2%). As suggested by this analysis, the supply of occupations typical for men tends to be in the direction of a prestige polarization whereas the occupational structure for women moves in the direction of prestige upgrading.

The vast increase of women in high prestige occupations challenges the male dominance in “the best occupations” and thus the mere dynamics of vertical segregation (Charles and Grusky, 2005). Still, as the Swedish labor market, as in several other Western countries, suffers from a strong gender division of labor, these changes of the occupational prestige structure need to be analyzed also in combination with the horizontal gender segregation (see Table 1).

**Table 1:** Percentage of employees within each quintile, and share of women within the occupation of the five largest occupations within quintiles and in the top-prestige occupations, 1997 and 2015.

**Prestige points**	**Prestige points for the five largest occupations in each quintile**	**Share of employees within quintile (%)**			**Share of females within occupation (%)**		
		**1997**	**2015**	**Diff**.	**1997**	**2015**	**Diff**.
	**Quintile 1**					
27	Personal care and related workers (excl. child-care)	35.4	33.8	−1.6	89.1	81.1	−8.0
21	Helpers and cleaners in offices, hotels and other establishments	16.2	12.9	−3.3	85.3	70.8	−14.5
28	Shop salespeople and demonstrators	13.3	21.9	8.6	55.6	60.1	4.5
23	Child-care workers	9.5	8.6	−0.9	95.3	82.7	−12.6
25	Building caretakers	5.2	4.0	−1.2	9.2	11.8	2.6
	**Quintile 2**						
37	Stock clerks	9.7	7.1	−2.6	19.1	24.6	5.5
37	Carpenters and joiners	7.1	9.4	2.3	0.8	1.1	0.3
32	Motor-vehicle drivers (excl. heavy-truck and lorry drivers)	5.3	8.0	2.7	3.2	10.7	7.5
31	Chefs	5.2	7.2	2.0	61.1	47.0	−13.1
33	Heavy-truck and lorry drivers	5.2	9.1	3.9	2.0	5.3	3.3
	**Quintile 3**						
40	Other office clerks	17.0	10.0	−7.0	87.5	79.1	−8.4
44	Nursing associate professionals	7.3	9.9	2.6	92.6	90.5	−2.1
38	Machine-tool operators	6.7	5.6	−1.1	10.7	8.7	−2.0
41	Accounting and bookkeeping clerks	6.3	11.0	4.7	90.5	88.0	−2.5
42	Agricultural or industrial machinery mechanics and fitters	5.4	3.4	−1.0	3.7	2.3	−1.4
	**Quintile 4**						
46	Technical and commercial sales representatives	11.9	13.9	2.0	23.2	30.2	7.0
50	Pre-primary education teaching associate professionals	11.5	11.6	0.1	92.1	90.2	−1.9
46	Mechanical engineering technicians	7.1	6.4	−0.7	6.5	11.1	4.6
51	Computer systems designers, analysts, and programmers	6.7	13.0	6.3	22.9	20.7	−2.2
49	General managers in wholesale and retail trade	6.6	2.5	−4.1	33.4	42.3	8.9
	**Quintile 5**						
57	Primary education teaching professionals	9.6	7.4	−2.2	77.4	77.7	0.3
60	Secondary education teaching professionals	7.4	5.0	−2.4	51.5	55.6	4.1
57	Business professionals not classified elsewhere	5.7	9.0	3.3	36.4	45.4	9.0
54	Nursing and midwifery professionals	5.5	3.6	−1.9	92.4	87.8	−4.6
58	Authors, journalists, and other writers	4.1	3.4	−0.7	44.2	55.0	10.8
**Prestige Points**	**Five occupations with the highest prestige points (quintile 5)**	**Share of employees within quintile (%)**			**Share of females within occupation (%)**		
		**1997**	**2015**	**Diff**.	**1997**	**2015**	**Diff**.
78	University, higher education teaching professionals	3.6	2.7	−0.9	37.6	44.9	7.3
78	Medical doctors	3.5	3.6	0.1	43.8	56.9	13.1
76	Judges	0.2	0.4	0.2	33.6	64.6	31.0
75	Physicists and astronomers	0.1	0.2	0.1	42.1	36.3	−5.8
74	Lawyers	0.5	0.5	0	9.6	41.4	31.8

*Weighted LFS data*.

**Figure 2:**
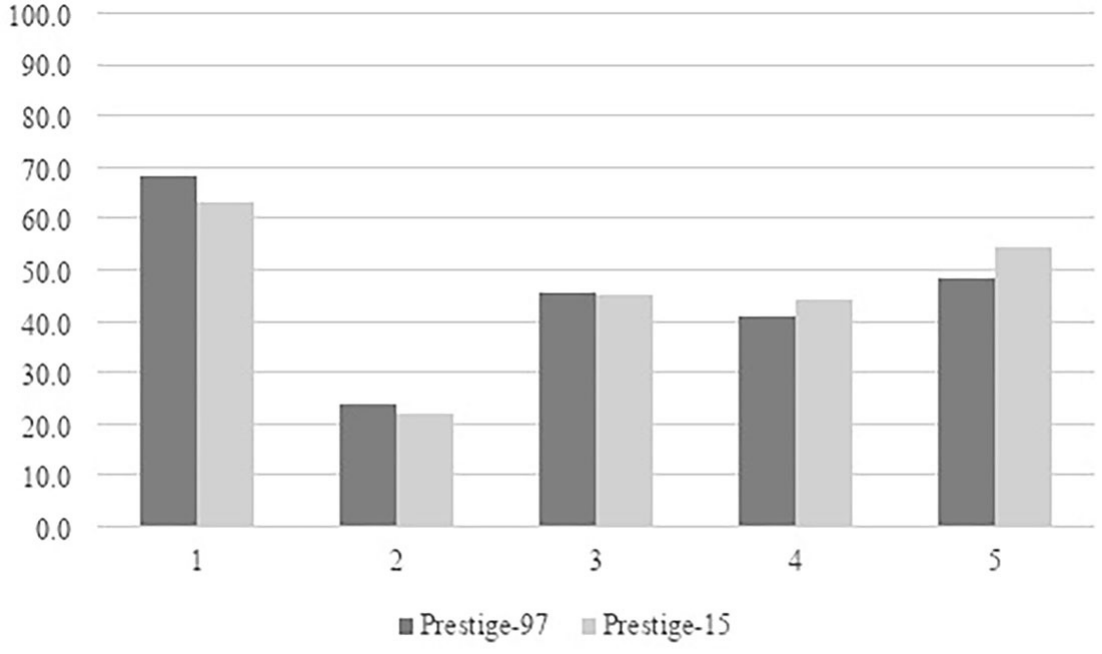
Share of women in quintiles 1–5, 1997 and 2015 (percentage). Weighted LFS-data.

As illustrated in Figure 2, in 1997, the lowest prestige group had 68 percent women, and by 2015 this proportion had declined to 63 percent. The vast majority of employees in low-prestige occupations are, consequently, still women. Prestige group 2 was male-dominated, with 24 percent women in 1997, and a slight decrease of women in 2015. Thus, the lowest prestige groups continue to be highly sex-segregated, upholding the horizontal dynamic (Charles and Grusky, 2005).

The number of women increased in the two highest prestige groups – nearly 6 percentage points in prestige group 5 and about 3 percentage points in group 4. Using the 40/60-percent criteria, women and men are numerically equal in the three highest prestige groups on the labor market, and gender segregation is primarily challenged in upper prestige groups. As women have increased in higher education (Statistics Sweden, 2018), this is somewhat expected.

### Gender Segregation Within Prestige Groups

The share of women in high-prestige groups has increased, but keeping the horizontal sex segregation in mind, a more detailed examination of each prestige group is required. The analysis below (Table 1) focuses on changes in the share of women in the five largest occupations within each prestige group as well as in the five most prestigious occupations.

In almost all of the occupations analyzed, the single-sex domination decreased between 1997 and 2015. Helpers and cleaners, child-care workers, and chefs showed the greatest changes, but even though the number of men increased in the first two, they continued to be strongly female-dominated, while chefs became a sex-equal occupation due to the increase in the share of men. Several of the occupations in prestige groups 1–2 are typically low-skilled non-routine jobs that, according to the RBTC theory, are less affected by technological changes (Autor et al., 2003).

In the middle-prestige group, technological advances have had some impact. *Office clerk* is a typical representative of an occupation where work tasks can be replaced with new technology. Accordingly, there is a rather strong decline (-7 percentage points) that has affected the sex composition in an equalizing way, although the occupation is still female-dominated (79%). The middle prestige group contains strongly sex-segregated occupations with few signs of change.

In prestige group 4, women increased in occupations related to technical and commercial sales and in wholesale/retail management. *Computer system designers, analysts*, and *programmers –* occupations at the core of digitalization – increased by 6.3 percentage points. However, this increase has not been advantageous for women, with a 2 percentage points decrease in employment.

Within the highest-prestige group, as well as in the five top-ranked occupations, women have increased in all but two occupations. The share of women has increased among *business professionals* (from 36 to 45%), and they have become the majority among *authors, journalists, and writers* (55%). Among the highest-prestige occupations, substantial increases have taken place among *judges* (from 34 to 65 % women), *lawyers* (from 10 to 41%), and *medical doctors* (from 44 to 57%). Thus, high-prestige occupations seem to have become less sex-segregated than the other prestige groups, where some even move toward female dominance.

To sum up, women have advanced vertically in the prestige hierarchy, but the Swedish labor market remains horizontally sex-segregated, particularly in the low prestige occupations.

### Decomposition Analysis of the Mean Change of Occupational Prestige

The changes in the occupational prestige structure, described in the analyses above, suggested an increase in employment in occupations with higher prestige. This indicates changes in the overall mean value of prestige in the labor market. The mean level has increased 1.9 points on the prestige scale, from 40.6 in 1997 to 42.5 in 2015. To explore what structural changes that drive the overall changes of prestige between the years of study, a Blinder-Oaxaca decomposition was conducted. Besides sex, the decomposition included five independent variables expected to be of relevance for the prestige distribution (Tables 2, 3, columns 1–3).

**Table 2:** Blinder-Oaxaca decomposition of the change in occupational prestige between 1997 and 2015.

	**Mean values 1997**	**Mean values 2015**	**B_1997_**	**B_2015_**	**B_pooled_**	**Explained**	**Unexplained**
Occupational prestige	40.55	42.50					
Sex	0.48	0.50	0.36*	0.63***	0.46***	0.01*	0.13
Occupational wage structure	28.70	29.77	1.18***	1.17***	1.17***	1.26***	−0.54
Age	41.04	42.25	0.07***	0.05***	0.58***	0.07***	−0.85**
Education: tertiary	0.29	0.45	8.58***	7.76***	8.09***	1.24***	−0.30***
Origin/foreign-born	0.09	0.14	−0.96***	−2.11***	−1.66***	−0.08***	−0.13***
Sector: public	0.33	0.30	0.49**	2.89***	1.79***	−0.06***	0.76***
Year: 1997					0.49***		
Constant			0.95*	1.38***	0.93***		0.43
Total						2.44	−0.49
Overall						1.94	
*N*			14,252	17,340	31,592		

*Unweighted LFS-data only including rotation group 1*.

Significance level: *p < 0.05; **p < 0.01; ***p < 0.001.

**Table 3:** Blinder-Oaxaca decomposition of the change in occupational prestige for *men and women* in occupations *above* the median occupational prestige, 1997–2015.

	**Mean values 1997**	**Mean values 2015**	**B_1997_**	**B_2015_**	**B_pooled_**	**Explained**	**Unexplained**
**Men upper half**							
Occupational prestige	51.86	53.44					
Occupational-wage structure (1000ths SEK)	34.54	35.59	0.65***	0.63***	0.64***	0.67***	−0.55
Age	42.49	43.16	0.04***	0.01	0.02***	0.02*	−1.02*
Education: tertiary	0.44	0.60	3.88***	3.33***	3.59***	0.56***	−0.28
Origin: foreign-born	0.08	0.10	0.53	0.81**	0.70**	0.02*	0.02
Sector: public	0.20	0.20	6.52***	6.44***	6.50***	0.03	−0.02
Year: 1997					−0.28***		
Constant			24.79***	26.91***	26.07***		2.12**
Total						1.30	0.28
Overall						1.58	
*n*			3,557	4,348	7,905		
**Women upper half**							
Occupational prestige	52.06	53.76					
Occupational-wage structure (1000ths SEK)	31.35	32.65	0.71***	0.68***	0.69***	0.90***	−0.98
Age	42.22	43.54	0.03**	0.01	0.02**	0.02*	−0.63
Education: tertiary	0.65	0.76	3.05***	2.86***	2.97***	0.35***	−0.14
Origin: foreign-born	0.08	0.12	0.77*	0.79**	0.78**	0.03**	0.00
Sector: public	0.55	0.50	3.48***	3.18***	3.29***	−0.17***	−0.16
Year: 1997					−0.56***		
Constant			24.63***	27.10***	26.40***		2.46*
Total						1.14	0.56
Overall						1.70	
*n*			3,067	4,790	7,857		

Significance level: *p < 0.05; **p < 0.01; ***p < 0.001.

The increase of the overall mean in prestige shown in Table 2, from 40.6 in 1997 to 42.5 in 2015, is mainly explained by two variables: the OW-structure and education. As discussed in the method section, the OW-structure has been used as a crude indicator of skills in demand in the labor market and strongly relates to technological changes (Autor et al., 2003). The mean of this variable increased from 28.70 to 29.77, indicating that employment in better-paid occupations increased between the 2 years. Similarly, the proportion of tertiary-educated in the labor market also increased from 0.29 to 0.45 (i.e., from 29 to 45%). The decomposition analysis showed that both of these compositional changes explained much of the change in the overall mean prestige over time (column 7). Of the estimated change of the model (2.44 prestige points), the OW-structure explained 1.26 and education 1.24 prestige points, respectively. This confirms the interrelatedness between prestige, education, skills, and wages (Carlsson, 1958; Reiss, 1961; Treiman, 1977; Ulfsdotter Eriksson, 2006).

The other independent variables (sex, origin, and sector) were less important for the overall increase in the overall mean value of prestige. However, the unexplained part of the decomposition (column 8) reveals that age and sector as impact factors have changed considerably. The coefficients indicate that the difference in mean-prestige between public and private employed had grown larger, with increasing numbers of employees in higher prestige occupations in the public sector, or alternatively, higher numbers of employees in low prestige occupations in the private sector (columns 4 and 5). The decreasing positive impact of age implies that over time it has become weaker related to individuals' placement in the prestige distribution.

The decomposition analysis revealed that changes in the sex composition of the labor force do not explain differences in mean prestige between 1997 and 2015. However, the analysis showed a stronger polarized pattern for men than for women, implying dissimilar processes of the distribution of occupational prestige. To study those processes, separate decompositions for men and women were conducted for the upper and lower halves of the prestige distribution (cut at the median prestige). Table 3 firstly presents the analysis of mean change for the upper half of the prestige distribution separately for men and women.^1^

In the upper half, the mean occupational prestige increased by 1.5 (from 51.9 to 53.4) for men, and by 1.7 (from 52.1 to 53.8) for women. For both sexes, the main explanatory factors were changes in the OW-structure and education. The OW-structure explained approximately 53 percent of the mean change for women, compared to 42 percent for men. This is an effect of the stronger increase of women in high-wage occupations (an increase in mean values by 1.30) compared to the somewhat lower increase for men (1.05). This finding supports previous research on declining sex segregation in higher white-collar occupations (Emerek, 2008; Halldén, 2014). Still, the mean level of the OW-structure was higher for men (35.59) than for women (32.65).

Tertiary education was a stronger explanatory factor of mean change for men (35%) than for women (21%). The share of men with tertiary education increased strongly during the period, without reaching the level of highly educated women. Thus, over time higher education has become a more decisive factor for men to reach high-prestige occupations, while for women this has more or less always been the case.

An additional explanatory factor, only applicable for women, was the reduced size of public sector employment in the upper half of the prestige distribution (from 55 to 50%). The reduction contributed negatively, meaning that the decrease in public sector employment has held back increases in the level of prestige for women. This phenomenon is explained by the generally higher prestige in the public sector in the upper half of the prestige distribution. Among men, the difference in prestige between the sectors was even larger (6.5 compared to 3.3 among women), indicating that, in the public sector, more men than women were found in high-prestige occupations. However, the absence of change in the share of men (20%) shows why this factor had no explanatory power.

The age effect for men has changed considerably over time. The strong positive impact of age on prestige in 1997 implied that the prestige distribution was strongly and positively structured by age, while no significant impact was found in 2015. Consequently, this change in the size of the coefficients implies a rather large unexplained part, while the increasing mean age in time did not contribute much to the overall change in the mean of the prestige distribution. A similar pattern also exists for women.

Turning to the lower half of the prestige distribution (Table 4), it is notable that the average prestige point was higher for men than for women (both years), and that the average prestige decreased for both sexes, albeit more for men (−0.8) than for women (−0.4). For men, there are no decisive explanatory factors; instead, large estimates were found for the unexplained part, which indicates that the B-coefficients have changed considerably over time. This relates particularly to the OW-structure, where the mean value decreased weakly between 1997 and 2015, while the coefficient increased from 0.6 to 0.9. The latter change rendered a strong positive (unexplained) effect on the difference (7.8). For women, the effect of the OW-structure was positive both years, and as the mean-level of the OW-structure slightly increased (from 23.80 to 23.96), the change works in the direction of an increase in the prestige level. This positive effect, however, was strongly counteracted by the reduced impact of the OW-structure on the prestige distribution at the latter time-point. Consequently, a strong negative component was found in the unexplained part of the decomposition.

**Table 4:** Blinder-Oaxaca decomposition of change in occupational prestige for *men and women* in occupations *below* the median occupational prestige, 1997–2015.

	**Mean values 1997**	**Mean values 2015**	**B_1997_**	**B_2015_**	**B_pooled_**	**Explained**	**Unexplained**
**Men lower half**							
Occupational prestige	32.22	31.41					
Occupational-wage structure (1000ths SEK)	26.01	25.91	0.60***	0.90***	0.75***	−0.07	7.84***
Age	39.62	41.07	0.04***	0.03***	0.03***	0.05***	−0.44
Education: tertiary	0.09	0.17	0.25	0.11	0.15	0.01	−0.02
Origin: foreign-born	0.10	0.17	−1.11***	−0.86***	−1.00***	−0.07***	0.03
Sector: public	0.13	0.11	−1.99***	−1.74***	−1.92***	0.05**	0.03
Year: 1997					0.79***		
Constant			15.39***	7.16***	10.78		−8.23***
Total						−0.03	−0.79
Overall						−0.82	
*n*			3,830	4,369	8,199		
**Women lower half**							
Occupational prestige	29.07	28.66					
Occupational-wage structure (1000ths SEK)	23.80	23.96	1.66***	1.22***	1.46***	0.23***	−10.45***
Age	40.17	40.96	0.05***	0.03***	0.04***	0.03*	−0.89*
Education: tertiary	0.08	0.20	1.55***	1.24***	1.26***	0.16***	−0.03
Origin: foreign-born	0.11	0.18	−0.92**	−1.72***	−1.32***	−0.09***	−0.11*
Sector: public	0.48	0.38	−3.59***	−2.17***	−2.92***	0.31***	0.61***
Year: 1997					1.04***		
Constant			−10.70***	−0.87	−6.82***		9.83***
Total						0.63	−1.04
Overall						−0.41	
*n*			3,798	3,833	7,631		

Significance level: *p < 0.05; **p < 0.01; ***p < 0.001.

Besides the significance of the OW-structure in the lower half of the prestige distribution, the impact among women of several of the other variables is noteworthy. Most of them implied an increase in prestige over time relating to age, education, and sector. The (slight) increase in mean age, and the increasing share of tertiary educated, should lead to positions of higher prestige. Regarding the sector, there was a negative relationship, which was reversed compared to the effect in the upper half of the prestige distribution (Table 3). Consequently, a decrease in the share of women working in the public sector (from 48 to 38%) implies increased prestige over time. However, most of these expected compositional effects in a positive direction are overrun by the unexplained part; in particular, the changing effect of the OW-structure discussed above. The only variable that contributes for both sexes as an explained effect of decreased prestige is the share of foreign-born workers, which share has increased over time.

## Concluding Discussion

Previous research on changes in the occupational job structure in Sweden has reached different conclusions, where some studies, focusing on skills, show continuously upgrading (Tåhlin, 2019; cf. Oesch and Piccitto, 2019), whereas those analyzing the wage structure find polarization (Åberg, 2015; Adermon and Gustavsson, 2015; Heyman, 2016). The present study contributes to this research by analyzing changes in the occupational job structure by exploring occupational prestige in Sweden at two points of time, 1997 and 2015. The arguments for using occupational prestige as the dependent variable were twosome: First, the occupational prestige hierarchy has proved a stable measure of occupations social standing over time (Treiman, 1977; Nakao and Treas, 1994; Svensson and Ulfsdotter Eriksson, 2009). Second, as a multidimensional concept, occupational prestige captures the variables used in explaining changes in the job structure such as skills, education, and wages. In addition, this study applied a gender perspective to the question and analyzed how changes had affected how women and men are distributed vertically and horizontally, and what that may explain changes in occupational prestige mean scores.

Looking at the employment patterns from a prestige perspective, the overall patterns showed tendencies of polarization as the lowest and highest prestige groups had grown and the middle prestige group had declined. However, the gender analysis showed that the changes had affected women and men differently. Women's employment showed signs of upgrading as they to a larger degree have entered high-prestige and high-wage occupations. Men were more subjected to job polarization, with an increase of employment in low-prestige and low-wage occupations and high-prestige high-wage ones as well.

The distribution of women and men over the occupational prestige hierarchy has changed vertically and horizontally between the studied points in time. The increase of employment in high-prestige occupations has unequivocally been beneficial for women, who have increased in share and numbers in the most prestigious occupations. In 2015, the highest prestige group was slightly female-dominated and among the five most prestigious occupations, the number of women increased in all but one. Three of the top-prestige occupations have become numerically gender-equal, and one is even dominated by women. Altogether, these findings challenge male dominance in high-prestige occupations, as well as the saying that the best occupations are positioned by men (cf. Charles and Grusky, 2005).

The strong sex segregation in the low-prestige occupations has endured, but some occupations have become somewhat more gender-equal due to an increase of men in female-dominated occupations. In line with previous research, it seems harder to break the sex segregation in the lower strata of the prestige hierarchy (England, 2010; Torre, 2019). The findings suggest that men have increased their share in low-prestige jobs that also are relatively low-paid, such as personal service jobs (Storm et al., 2017). In general, women may not be equally affected by these changes as they are often found in service sectors that are growing. Women from working-class backgrounds tend to opt for upward mobility by turning to service or clerical occupations rather than male-dominated blue-collar occupations (England, 2010).

Top-prestige occupations were previously dominated by men (England 1979), whereas the present study showed that the proportion of women has increased in high-prestige occupations over time, making women the majority in the higher prestige strata. Thus, it no longer seems reasonable to claim that “the best” occupations are dominated by men (Charles and Grusky, 2005) – if by “best” we refer to the most prestigious ones. Women's equal amount within the upper prestige strata challenges the notion of men being at the top of the vertical segregation. It is also interesting to note that men are entering low-prestige and female-dominated occupations. This study shows tendencies of decreasing sex segregation, also confirmed in previous research (Emerek, 2008; Bihagen et al., 2014; Halldén, 2014). In this sense, we are moving toward increased gender equality.

Still, the changes occurring seem to be especially beneficial for women in the upper end of the strata. The lower half showed decreasing prestige for both sexes, and more so for men. These findings can be explained by changes in the occupational structure, where blue-collar and male-dominated industrial work is decreasing. Those jobs were often well paid but had low prestige (Tåhlin, 2019). Instead, men have increased their share in low-prestige jobs that are relatively low-paid, such as personal service jobs. In general, women may not be equally affected by these changes as they are often found in service sectors. However, during the period under study, women made intrusions into typical male-dominated occupations. These are of relatively low prestige, but often better paid than traditional service work.

The occupational prestige mean score increased from 1997 to 2015, indicating that a larger number of employees are within high-esteem occupations, thus supporting the notion of an upgrading tendency in the occupational structure (Oesch and Piccitto, 2019; Tåhlin, 2019). More importantly, and as shown by the decomposition analysis, changes in the wage structure and education/qualifications contributed nearly equally to the increase. Especially, the up-grading of the occupational-wage structure has been particularly beneficial for women entering high prestige jobs. Generally, women's wages have improved, and the wage gap decreased from 22 percent in 1996 to 11 percent in 2016 (Statistics Sweden, 1998; SNMO, 2017). Moreover, the larger share of women in tertiary education has proven beneficial regarding both high-end employment and prestige distribution. Higher education had a stronger explanatory factor for men than for women suggesting that education over time has become important also for men to be qualified for employment in high-prestige occupations.

The analysis also indicated that the sector of employment affected changes in the prestige mean. The public sector in Sweden, traditionally dominated by women, has changed a lot during the period (Berglund and Esser, 2014). The public sector strongly declined during the 1990s crisis in Sweden and child-, elderly and personal care has to a larger extent been privatized resulting in a transfer of low-paid occupations to the private sector. In general, the public sector has higher mean prestige than the private sector. At the same time, however, the public sector offers jobs in both ends of the prestige distribution to a higher extent than the private sector. Consequently, the downsizing of the sector had paradoxical consequences for female prestige placement. On the one hand, had the strong decline of females in low-prestige jobs in the sector a positive impact on their overall prestige. On the other hand, the reduction of female employment in the upper half of the prestige distribution reduced this positive effect. For males were these tendencies not equally strong.

In conclusion, we see overall equalizing tendencies in the distribution of occupational prestige, foremost in the upper end of the distribution. However, the results do not only point in a positive direction—toward upgrading (Oesch and Piccitto, 2019; Tåhlin, 2019). The findings showed polarization tendencies among men, and by that also partly confirming Åberg (2015). The implications of a polarization tendency among men, parallel to an upgrading pattern for women is an important empirical question for future research, beyond the scope of this article. Nevertheless, what our results have especially highlighted is that occupational prestige is an important piece of the puzzle that needs to be taken into account in the study of occupational change.

## Data Availability Statement

The data analyzed in this study is subject to the following licenses/restrictions: confidentiality on register data. Requests to access these datasets should be directed to Tomas.Berglund@socav.gu.se.

## Author Contributions

All authors listed have made a substantial, direct, and intellectual contribution to the work and approved it for publication.

## Funding

This study was funded by Forte, Swedish Research Council for Health, Working Life and Welfare (Dnr 2016-07204).

## Conflict of Interest

The authors declare that the research was conducted in the absence of any commercial or financial relationships that could be construed as a potential conflict of interest.

## Publisher's Note

All claims expressed in this article are solely those of the authors and do not necessarily represent those of their affiliated organizations, or those of the publisher, the editors and the reviewers. Any product that may be evaluated in this article, or claim that may be made by its manufacturer, is not guaranteed or endorsed by the publisher.

## Footnotes

^1^A separate decomposition analysis for the upper and lower halves, including both women and men, can be requested from the authors. The results of this analysis show that sex *per se* is not an explaining factor in terms of difference in mean values between 1997 and 2015.
